# Chemotherapy-Induced Peripheral Neuropathy: A Recent Update on Pathophysiology and Treatment

**DOI:** 10.3390/life14080991

**Published:** 2024-08-09

**Authors:** Marina Mattar, Florence Umutoni, Marwa A. Hassan, M. Wambui Wamburu, Reagan Turner, James S. Patton, Xin Chen, Wei Lei

**Affiliations:** 1 Department of Pharmaceutical and Administrative Sciences, Presbyterian College School of Pharmacy, Clinton, SC 29325, USA; mmattar@presby.edu (M.M.); mhassan@presby.edu (M.A.H.); 2 Department of Pharmaceutical and Graduate Life Sciences, College of Health Sciences, Nursing, and Pharmacy, Manchester University, Fort Wayne, IN 46845, USA; fumutoni2025@manchester.edu (F.U.); jspatton2027@manchester.edu (J.S.P.); 3 Department of Pharmacy Practice, College of Health Sciences, Nursing, and Pharmacy, Manchester University, Fort Wayne, IN 46845, USA; mwwamburu@manchester.edu; 4 Department of Biology, Presbyterian College, Clinton, SC 29325, USA; rbturner@presby.edu; 5 Department of Pharmaceutical and Clinical Sciences, College of Pharmacy and Health Sciences, Campbell University, Buies Creek, NC 27506, USA; xchen@campbell.edu

**Keywords:** chemotherapy-induced peripheral neuropathy, pathophysiology, duloxetine, cannabinoid, pain

## Abstract

Chemotherapy-induced peripheral neuropathy (CIPN) is a major long-lasting side effect of some chemotherapy drugs, which threatens cancer survival rate. CIPN mostly affects sensory neurons and occasionally motor neurons, causing numbness, tingling, discomfort, and burning pain in the upper and lower extremities. The pathophysiology of CIPN is not completely understood; however, it is believed that chemotherapies induce peripheral neuropathy via directly damaging mitochondria, impairing the function of ion channels, triggering immunological mechanisms, and disrupting microtubules. The treatment of CIPN is a medical challenge, and there are no approved pharmacological options. Currently, duloxetine and other antidepressants, antioxidant, anti-inflammatory, and ion-channel targeted therapies are commonly used in clinics to relieve the symptoms of CIPN. Several other types of drugs, such as cannabinoids, sigma−1 receptor antagonists, and nicotinamides ribose, are being evaluated in preclinical and clinical studies. This paper summarizes the information related to the physiology of CIPN and medicines that could be used for treating this condition.

## 1. Introduction

Chemotherapy-induced peripheral neuropathy (CIPN) is defined as an injury of the somatosensory nervous system after treatment with chemotherapy [1]. There are two types of sensory abnormalities contributing to the development of motor and autonomic changes in CIPN: these include abnormal sensory discrimination of touch, vibration, and thermal information and a tingling or burning sensation also referred to as mechanical allodynia [2]. The severity and the duration of CIPN depends on, treatment agent, chemotherapy dose, duration, a combination of neurotoxic drugs, and the presence of preexisting diseases such as diabetes or chronic kidney disease [3]. In most cases, the sensation happens in the hands and feet known as “gloves and stocking” distribution [4]. These symptoms can happen with just one high single dose or after accumulation of chemotherapy drugs [1]. Symptoms usually stabilize during treatment and relieve with time after termination. However, about one-third of patients experience symptoms after 6 months of treatment cessation [5]. In some cases, the chemotherapy drugs can persist in the nerve axon after treatment and worsen the neuropathic symptoms, and this is known as the “Coasting Phenomena” [3,6].

CIPN is a serious problem that ranks as a major long-lasting adverse effect related to chemotherapy medications, such as platinum, ixabepilone, and thalidomide [7,8]. It is estimated that 30–40% of patients undergoing chemotherapy will experience CIPN each year and can lead to either premature treatment termination or chemotherapy dose reduction that influences the treatment efficacy and survival rate [9,10]. The prevalence range of CIPN is from 19% to more than 85%. The severity of CIPN depends on the length and agent used with some chemotherapeutic agents having the highest rates such as platinum agents, taxanes, vinca alkaloids, proteasome inhibitors, and immunomodulatory drugs [11]. Seventy percent of patients who received paclitaxel and 90% of those who received oxaliplatin develop the symptoms of CIPN [12].

The treatment of CIPN has been a medical challenge. The foremost challenge is the lack of agents recommended for CIPN prevention and management in the American Society for Clinical Oncology clinical practice (ASCO) guidelines [13]. Current recommendations suggest a focus on symptoms affecting quality of life by using pharmacological interventions for pain and physiotherapy solutions like exercise, acupuncture, massage therapy for functional impairment, and in some cases provision relief [14]. However, despite current efforts, CIPN is still not well managed in the majority of patients and has a negative impact on quality of life [13]. Duloxetine is the only off-label FDA-approved drug that has shown efficacy in treating CIPN when compared to antidepressants and anticonvulsants [13,15]. In addition, CIPN also increases the economic burden, while decreasing quality of life (QOL) [13]. A study carried out where both physical and mental health were evaluated to measure QOL showed that a higher symptom burden is associated with a lower QOL [16]. On cost, a 2022 review of studies carried out on CIPN treatment indicated that the monthly drug cost of treating CIPN has a range of USD 15 to USD 1425 [17]. Further, the symptoms of CIPN reduce work efficiency resulting in loss of job, which adds to the economic burden [2].

Treating CIPN is a challenge due in part to the prioritization of life-threatening chemotherapy side effects over CIPN, discussions of side effects are more centered around acute issues like nausea, vomiting, hair loss, and infections [18]. Even though there are advances in understanding the biological mechanisms of chemotherapy-induced peripheral neuropathy, few prevention or treatment options exist [10,15]. Therefore, discovering efficient approaches for CIPN management is highly demanding.

In this paper, we will discuss the pathology of CIPN and summarize the current pharmacological therapy utilized in CIPN. Further, the medicines under clinical and preclinical investigation will be discussed. We also summarize the recent findings of the mechanism of action, safety, and efficacy of drugs for treating CIPN. In addition, this paper will list some limitations and future directions related to these options based on data from clinical trials. 

## 2. Pathophysiology of Chemotherapy-Induced Peripheral Neuropathy

Chemotherapy medications induce peripheral neuropathy via different mechanisms: changes in neuronal cytoskeleton, directly damaging neuron/DRG mitochondria, impairing the function of ion channels, triggering immunological mechanisms via glial cell activation, and disrupting microtubules [4].

### 2.1. Cytoskeleton Changes

A functional cytoskeleton is required to maintain the function and integrity of the nervous system [19]. Chemotherapy medications promote major modifications in the neuronal cytoskeleton, resulting in the fragmentation of axons in sensory neurons leading to changes in the microtubule network [19,20]. These changes compromise the structural integrity of sensory neurons, resulting in impaired axonal transport of mitochondria and mRNA [21]. For example, paclitaxel and vincristine can cross the blood-nerve-barrier and bind to beta-tubulin of sensory nerve fibers where they affect the structure of the cytoskeleton of healthy peripheral neurons, such as the primary afferent neurons of the dorsal root ganglia [19,20]. 

### 2.2. Oxidative Stress 

Mitochondria are membrane-bound cell organelles responsible for producing energy through the oxidative phosphorylation process [22]. Chemotherapy can cause mitochondrial dysfunction through different mechanisms [23]. Platinum binds to mitochondrial DNA and impairs replication, transcription, and protein synthesis, resulting in respiratory chain damage, reduction of cellular metabolism, and increased oxidative stress which leads to damage to peripheral nerves [24,25,26,27,28,29,30,31]. Taxanes can alter mitochondrial membrane permeability pores leading to the release of calcium, which further activates calpain that catalyzes protein degeneration including neuronal calcium sensor 1 (NCS1). These changes lead to mitochondrial depolarization, impaired energy production, and neuronal dysfunction which plays a role in the induction of neuropathic pain [32].

### 2.3. Ion Channel

Altered expression of the voltage-gated sodium channel (NaV), voltage-gated potassium channel (Kv), and voltage-gated calcium channel (CaV) leads to a change in neuronal excitability [33,34]. NaV is essential to initiate and propagate action potential in neurons [25]. Chemotherapeutic drugs can increase the expression of NaV in nociceptive neurons increasing action potential [34]. The amplification of action potential in nociceptive neurons results in hyperexcitability and spontaneous firing of neurons which results in increased pain sensitivity and neuropathic pain [35]. Treatment with paclitaxel increases the expression of NaV in DRG causing increased action potential firing resulting in hyperexcitability [36,37]. Significant downregulation of voltage-gated potassium channels has been found in patients after oxaliplatin treatment, leading to increased membrane excitability in neurons [38,39]. Chemotherapy can also upregulate voltage-gated calcium channels that change peripheral neurons’ action potential threshold, causing hyperexcitability [40,41,42].

### 2.4. Triggering Immunological Responses

Previous studies have demonstrated a strong correlation between CIPN and increased levels of pro-inflammatory cytokines. This is because cytokine signaling, crucial for neuroinflammation and sensitization in the sensory nervous system, plays a key role in chemotherapy-induced peripheral neuropathy (CIPN) [33]. CIPN is characterized by increased levels of pro-inflammatory cytokines (TNF-α, IL-1β, IL-6) and reduced levels of the anti-inflammatory cytokine IL-10 [36,43]. Oxaliplatin can activate astrocytes leading to the activation of the pro-inflammatory cascade and production of pro-inflammatory cytokines such as IL-Iβ, TNF-α, and IL-6 [44,45,46]. Taxanes can bind to toll-like receptor 4 (TLR-4) triggering activation of NF-κB [42].

### 2.5. Disruption of Microtubules 

Taxanes and Vinca Alkaloids also induce peripheral neuropathy by interrupting tubulin polymerization. After binding to tubulin, Taxanes and Vinca Alkaloids cause microtubule dysfunction leading to alteration of axonal synaptic vesicle transportation as well as interfering with the regeneration and remodeling of axons [47,48,49,50]. 

## 3. Current Approach for CIPN Management 

Based on the most up to date ASCO guidelines, there is no agent recommended for the prevention of CIPN [13]. However, the ASCO does recommend that patients with CIPN should receive reduced or delayed doses and substitute or terminate the chemo agent [13]. Duloxetine (brand name Cymbalta), an agent off-label used to treat CIPN, is the only agent with moderate efficacy [13,51]. Duloxetine inhibits serotonin and norepinephrine reuptakes, which increases the availability of the key neurotransmitters to activate the descending pathway [52]. Duloxetine could also reduce inflammation and nerve injury by inhibiting the activation of p38 and NF-kB [53]. Duloxetine has been tested in many clinical trials and has been approved to reduce pain compared with placebo and other classes of medications [29,41]. 

Several complementary and alternative medicines have been tested for treating CIPN. Nonpharmacological approaches, including acupuncture, exercise, mindfulness practices, yoga, meditation, and touch therapies like acupressure, reflexology, and massage, have been found to reduce chemotherapy-related symptoms and improve quality of life [54]. In addition, some nutrients and Chinese herbal medicines have shown potential therapeutic effects in patients with neuropathic pain [14].

There are major limitations in the current treatment of CIPN. A major limitation of duloxetine use is that insurance companies recommend using either pregabalin or gabapentin before the usage of duloxetine even though that is contradictory to the recommendation of ASCO guidelines [13]. By far, none of the complementary and alternative approaches has shown promising results for treating CIPN [54]. In addition, there is a lack of guidelines for using nutrients and herbs for the treatment of CIPN in clinics.

## 4. Medications under Clinical Trials

With no effective therapies to treat CIPN, several clinical trials have been carried out and continue to be carried out to find a clinical solution (Table 1). Therapies used for other disease states have been studied to ascertain their effectiveness in alleviating CIPN. However, we only include the clinical trials of which the results have been published. 

### 4.1. Serotonin and Norepinephrine Reuptake Inhibitors

Venlafaxine is a second-generation tricyclic antidepressant (TCA) that works by inhibiting neuronal serotonin activity, and norepinephrine and serotonin reuptake [55]. It is a good alternative for patients who cannot afford duloxetine [56]. A clinical trial conducted in 2018 found that venlafaxine effectively reduced the grade of cranial, motor, sensory, and neuropathic pain in the patients who had CIPN, but with less efficacy compared to Duloxetine [57]. In a phase III randomized double-blinded control trial, venlafaxine was effective in relieving acute neurotoxicity and improving functional status in patients who suffered from oxaliplatin-induced neuropathy [58]. However, in a pilot trial, Venlafaxine was not effective in preventing either acute or chronic neuropathy symptoms, such as throat discomfort and discomfort swallowing cold liquids, induced by oxaliplatin treatment [59]. Original guideline commentary for venlafaxine has shown its use as a preventative agent, however, longer follow-up data do not support this use [58]. 

Amitriptyline is a tricyclic TCA medication being tested in clinical trials [60]. One study showed that systemic treatment with amitriptyline was unable to relieve CIPN symptoms, including tingling, numbness, impaired sensory function, and pain in hands and feet [61]. In contrast, another clinical trial found that topical amitriptyline alone or with baclofen and ketamine effectively reduced the neuropathic pain associated with CIPN in patients who had received vinca alkaloids, oxaliplatin, cisplatin, taxanes, thalidomide or other neurotoxic agents [62,63]. Both amitriptyline and venlafaxine are not FDA approved nor have definite doses for treating CIPN [13,58,62]. In addition, they have more side effects compared to duloxetine. Thus, more trials need to be carried out to provide guidance on optimal doses with fewer side effects to treat CIPN [45]. 

### 4.2. Ion Channel Targeted Therapy 

Studies have shown that ion channel targeted therapies, such as lidocaine, gabapentin, and pregabalin, have also been successful in reducing CIPN [9]. Lidocaine is an antiarrhythmic drug that can non-selectively block sodium, potassium, and calcium channels [64]. Systemic treatment with lidocaine via intravenous injection was effective in pain reduction with an analgesic effect that persisted for an average of 23 days in patients [65]. This is further supported by a case where neuropathy symptoms were completely relieved by intravenous lidocaine infusion [66]. A recent randomized controlled study also found that lidocaine infusion showed a similar effect as duloxetine on reducing the incidence and severity of taxane-induced peripheral neuropathy [67]. 

Gabapentin and pregabalin are anticonvulsant agents that block presynaptic voltage-gated calcium channels and down-regulate excitatory [68]. Several clinical trials have evaluated the efficacy of gabapentin and pregabalin in CIPN prevention and treatment. A phase 3 randomized controlled trial found that treatment with gabapentin for 6 weeks failed to relieve the CIPN symptoms [69]. Another randomized trial also had similar results. Gabapentin (20 mg/kg/day) increased the pain scores and opioid consumption in pediatric patients with vincristine-induced neuropathy [70]. Treatment with pregabalin (75 mg) twice daily showed a trend to reduce numbness, but not any other paclitaxel-induced neuropathy symptoms [71]. More trials are needed to definitively provide indications of these drugs’ use in CIPN treatment [69].

### 4.3. Anti-Inflammatory Medications

Typically, these drugs are used in other disease states but also exhibit anti-inflammatory effects that have shown efficacy in decreasing CIPN. Metformin, a drug commonly used for treating diabetes, has shown anti-inflammatory effects on endothelial cells by inhibiting the production of IL-6 and TNF-α thus reducing the phosphorylation of mitogen-activated protein kinase (MAPK) [68]. Additionally, Metformin activates adenosine monophosphate-activated protein kinase (AMPK) which could inhibit the mammalian target of the rapamycin (mTOR) pathway causing nociception blockage [72]. A study was conducted using cisplatin and Paclitaxel, which are drugs known to induce mechanical allodynia. One arm had co-administration of Metformin with cisplatin and the other arm administered Metformin with Paclitaxel [45,72,73]. In a randomized controlled study, metformin 500 mg three times a day was given to patients receiving oxaliplatin in the FOLFOX-4 regimen for colorectal cancer. Metformin offered protection against oxaliplatin-induced chronic peripheral sensory neuropathy [74]. 

Minocycline is an antibiotic that inhibits the production of pro-inflammatory cytokines in monocytes and microglia to reduce mechanical sensitivity [75]. In the Academic and Community Cancer Research United (ACCRU) pilot study, Minocycline reduced the daily average pain score but no prevention of CIPN was found. However, when dosed based on weight, minocycline prevented chemotherapy-induced neuropathy in mice receiving paclitaxel [76,77]. In contrast, in a phase II randomized clinical trial where an oxaliplatin chemotherapy regimen was used, minocycline did not relieve fatigue or numbness/tingling symptoms and failed to reduce pro-inflammatory cytokines compared to placebo [78]. 

### 4.4. Antioxidant Medications

It has been shown that the antineoplastic agent oxaliplatin does induce peripheral neurotoxicity. One of the ways suggested on how this neurotoxicity occurs is oxidative stress, where there’s an increase of reactive oxygen species and reactive nitrogen species creating an imbalance between their production and removal. Mitochondrial superoxide dismutase (MnSOD) is what keeps the balance on these species. Mangafodipir, a contrast agent, was discovered to have mitochondrial superoxide dismutase mimetic activity and was therefore tested as a cytoprotectant [79].

In the PLIANT phase II placebo-controlled study, patients treated with oxaliplatin were given calmangafodipir (Pledox) which is derived from mangafodipir [80,81,82,83,84]. It was observed that there was a delay of onset and a reduction in intensity of the CIPN symptoms, in particular the cold allodynia symptoms, with calamangafodipir at a dose of 5 µmol/kg [83]. 

Amifostine is a potent free radical scavenger pro-drug and has been shown to protect healthy tissues during chemotherapy and radiation [85]. The active metabolite (WR-1065) demonstrated the prevention of oxaliplatin-induced neurotoxicity [86]. This protection is selective for non-tumor tissues [10,13]. However, in a phase II clinical trial, where cisplatin and 3-hour paclitaxel were the chemotherapy agents used [87]. Amifostine had a diminished effect on preventing CIPN [83]. These two trials demonstrate that amifostine is a strategy to be used specifically in oxaliplatin-induced neurotoxicity, however, it was not so in other platinum compounds like cisplatin and paclitaxel [83,87].

### 4.5. Sigma-1 Receptors (S1R)

Sigma-1 receptors (S1R) play an important role in modulating several types of ion channels such as NaV 1.2, NaV 1.4, KV 1.2, KV 1.3, and KV 1.4 [88,89]. They also can modulate intramitochondrial calcium homeostasis and trafficking of functional transient receptor potential ankyrin 1 (TRPA1), which play a critical role in the development of CIPN [90,91]. MR309 is a selective S1R antagonist that has shown efficacy in reducing allodynia and cold hypersensitivity [92]. A phase 1 trial found that MR309 has shown good safety and tolerability in healthy people [93]. In two other clinical studies, MR309 showed a significant reduction in cold allodynia and hyperexcitability motor symptoms compared to placebo [88,89]. 

### 4.6. Cannabinoids

The endocannabinoid system is an endogenous system responsible for pain perception modulation and has attracted interest in the management of CIPN [94]. It consists of two types of receptors, CB1 dominant in the brain and CB2 dominant in peripheral immune systems and central nervous systems [95]. Both CB1 and CB2 are G-protein coupled receptors and can modulate the activities of numerous intracellular signaling pathways, such as MAPK, phosphoinositide 3-kinase (PI3K)/Akt, Nrf2, and Ca^2+^-regulated signaling cascades [96]. Endocannabinoid system homeostasis is maintained via transporters that control the transport of anandamide (AEA) and 2-arachidonoylglycerol (2-AG) from the cells to synapse and vice versa [97]. The activity of the endocannabinoid system is also controlled by enzymes, such as fatty acid amino hydrolase, cyclooxygenase-2 (COX-2), and N-acylethanolamine-hydrolyzing acid amidase, which are involved in the degradation of the endocannabinoid [98].

The safety, efficacy, and pharmacokinetics of cannabinoids have been studied in several clinical trials [99,100,101,102,103,104,105]. A double-blind placebo-controlled study determined that oral mucosal spray containing cannabinoids had no significant difference in treating CIPN [106]. However, the five “responders” had 2.6 of 11-point NRS-PI lower than the placebo group [103]. Treatment with CBD oil (300 mg/daily) had lower scores on cold sensitivity to touch, discomfort swallowing cold liquids, and throat discomfort in patients with CIPN [105]. A case series reported that topical use of CBD alone or with THC reduced neuropathic pain in patients with CIPN [107]. Numerous clinical trials are ongoing to test the effect of cannabinoids on treating and preventing CIPN.

**Table 1:** Medications under clinical trials for the treatment of CIPN.

Intervention	Chemotherapy	Sample Size	Dose	Results	Reference
Amitriptyline		44	1 g of 10% amitriptyline cream twice a day	Topical amitriptyline was effective in reducing VAS pain score	[56]
Amitriptyline	Taxane, vinca alkaloid, or platinum	114	25 mg daily up to 100 mg daily	Amitriptyline was not effective in CIPN prevention	[61]
Amitriptyline and ketamine	Taxane	462	Apply 4 g of 2% ketamine plus 4% amitriptyline (KA) cream BID	No difference in 6-week pain score, numbness, and tingling	[63]
Amitriptyline Baclofen ketamine		208	baclofen 10 mg, amitriptyline HCL 40 mg, and ketamine 20 mg	BAK gel was statistically significant in motor neuropathy improvement but not statistically significant in sensory neuropathy	[62]
Amifostine	Cisplatin or paclitaxel	27	amifostine 740 mg/m^2^	Not effective in protection against CIPN	[87]
Calmangafodipir	Oxaliplatin	173	calmangafodipir 2 µmol/kg or 5 µmol/kg	Calmangafodipir can prevent CIPN during the 1st 6 months	[83]
Calmangafodipir	Oxaliplatin	592	Calmangafodipir 2 µmol/kg, or 5 µmol/kg, or placebo	Month 9 of treatment data shows 54% with moderate-severe CIPN and higher percentage(s) of hypersensitivity reactions	[84]
Cannabidiol	Carboplatin and paclitaxel, or capecitabine and oxaliplatin	54	150 mg CBD oil twice daily (300 mg/daily) for 8 days	Reduce cold sensitivity to touch, discomfort swallowing cold liquids, and throat discomfort	[105]
Cannabidiol		40	Topical use for 2 weeks	No improvement on painful established CIPN	[108]
Cannabinoid extract (Nabiximols)	Paclitaxel, vincristine, or cisplatin	16	Two to twelve sprays per day for a week	An average decrease of 2.6 on an 11-point NRS-PI in five “responders”	[106]
Capsaicin	Oxaliplatin	18	High dose 8% patch	Effective in treating pain associated with CIPN	[109]
l-carnosine	Oxaliplatin	65	L-carnosine 500 mg daily	Significant reduction in NF-κB and TNFα; Neuroprotective	[110]
Duloxetine		231	30 mg QD 7D, then 30 mg BID	Use for 5 weeks resulted in greater overall pain reduction	[51]
Duloxetine	Taxane	47	30 mg daily in the first week following the injection of paclitaxel and 60 mg during the second week	Reduces the scores of neuropathies, but not paresthesia, numbness, cold sensitivity, and other nerve conduction velocity (NCV) values	[111]
Gabapentin	Taxane, platinum, or vinca alkaloid	115	300 mg up to 2700 mg daily	Not effective in controlling CIPN symptoms	[69]
Gabapentin	Vincristine	49	20 mg/kg/day	Gabapentin was associated with more opioid consumption and high pain score compared to placebo	[70]
Glutamine	Vincristine	56	Glutamine 6 g/m^2^ BID	Well tolerated Sensory function and QOL improvement	[112]
Glutathione	Paclitaxel and carboplatin	185	1.5 g/m^2^ IV over 15 min immediately before chemotherapy	No significant difference in peripheral neuropathy	[113]
Glutathione and Mecobalamin		158	2.4 g of glutathione IV once daily 2–3 days before chemotherapy, plus 500 µg mecobalamin IV once every other day	Significant reduction in incidence and severity of CIPN	[114]
Huangqi Guizhi Wuwu Decoction (HGWD)	Paclitaxel	92	Washing limbs with HGWD for 20 min twice a day for consecutive 14 days	Largely reduces the CIPN sensory scores, but not the autonomic scores	[115]
Lafutidine	Carboplatin or paclitaxel	18	10 mg twice daily	Shows a trend to reduce neurotoxicity	[116]
Lamotrigine		131	Target dose 300 mg/day	Lamotrigine is not effective as CIPN treatment	[117]
Lidocaine		9	1.5 mg/kg for 10 min followed by 1.5 mg/kg/h over 5 h	Pain reduction that persists for 23 days average	[65]
Lidocaine	Taxane	60	2 mg/kg with saline infusion 40 min prior to taxane therapy	Decreased the incidence and severity of taxane-induced peripheral neuropathy	[67]
Lithium	Taxane	37	300 mg daily for 5 days	Not effective in CIPN prevention	[118]
*Melissa officinalis* (MO)	Cisplatin, oxaliplatin), vincristine, bortezomib, or taxanes	80	500 mg 2 times a day for 3 months	MO reduces pain and diarrhea based on the scores of EORTC QLQ-C30 (Integrated System for Quality of Life Assessment)	[119]
Metformin	Oxaliplatin	55	Metformin 500 mg TID	Less incidence of grade 2/3 neuropathy Significantly low pain score Low serum level of malondialdehyde and neurotensin High Ntx-12 score	[74]
Minocycline	Paclitaxel	47	200 mg on day 1 followed by 100 mg BID	Not effective in CIPN prevention, however, it is effective in *p*-APS score and fatigue reduction	[76]
Minocycline	Paclitaxel	66	100 mg BID	No significant reduction in fatigue/numbness symptoms with no difference in serum pro-inflammatory markers	[120]
Mirogabalin	Oxaliplatin or taxane	58	Between 5 and 15 mg twice daily	Reduce numeric rating scale (NRS) score	[121]
Mirogabalin	Taxane	43	5–30 mg daily	Relieve the CIPN symptoms	[122]
N-acetyl cysteine	Paclitaxel	75	1200 mg daily or 1200 BID	Significant reduction in peripheral neuropathy in high-dose group QOL, mTNS were significantly improved. Significant elevation of NGF serum level	[123]
Omega-3 fatty acids	Paclitaxel	60	4 g	No benefit	[124]
Pregabalin (children)		30	150–300 mg	Pregabalin improved pain symptoms significantly	[125]
Pregabalin	Paclitaxel	46	75 mg BID	Pregabalin not effective in reducing tingling pain, or EORTC QLQ-CIPN20 subscale scores	[71]
Pregabalin	Paclitaxel, docetaxel, or oxaliplatin	26	75 mg BID for 3 days, 150 mg BID for 3 days, then 300 mg BID until and including day 28	Pregabalin was not effective in reduction of average or worst pain compared to placebo	[126]
Pregabalin vs. duloxetine	Taxane	82	Pregabalin 150 mg once duloxetine 60 mg	Both were efficacious in decreasing CIPN	[127]
Renin-angiotensin-aldosterone system inhibitors	Paclitaxel	5886		Decreases the incidence of paclitaxel-induced CIPN in patients with lung cancer	[128]
Sigma-1 receptor antagonist	Oxaliplatin	124	400 mg MR309 daily dose during the first 5 days of each chemotherapy cycle	MR309 reduces sensory and motor hyperexcitability Patients able to get higher dose of oxaliplatin	[89]
Silybum marianum (SM)	Cisplatin	60	140 mg three times daily for 90 days	SM reduces the scores of DN4 (Douleur neuropathique 4 questions) and CIPNAT (chemotherapy-induced peripheral neuropathy assessment tool)	[129]
Venlafaxine	Oxaliplatin	50	37.5 mg XR BID	Not effective in prevention of either acute or chronic neuropathy	[59]
Venlafaxine	Oxaliplatin	48	50 mg 1 h prior oxaliplatin infusion then 37.5 mg BID from day 21 to 1	Effective in relieving acute neurotoxicity as well as improving functional status	[58]
Vitamin E	Taxane	140	Vit E 400 mg BID	Vit E is not effective in CIPN prevention; however, it helps with shortening of neuropathy	[130]

## 5. Preclinical Studies

Discovering novel treatments for CIPN has attracted a lot of interest since most of the medications tested in clinical trials are unsatisfactory. A meta-analysis conducted in 2015 determined that no drug showed promising effects in terms of preventing or treating CIPN [131]. Therefore, developing novel approaches is in high demand for improving the treatment of CIPN. Numerous compounds and targets have been tested in different animal models, even though these findings may not always be able to translate into patient care in clinics (Table 2).

### 5.1. Sphingosine-1-Phosphate (S1P) Receptor 1 Antagonist

Sphingosine-1-phosphate is a lipid that alters numerous cellular functions [132]. S1P binds to a group of G protein-coupled receptors (S1PR1-5), and increased activity of S1PR1 could contribute to the development of CIPN [133,134]. Fingolimod is a functional antagonist of S1PR1 and has been approved by the FDA for treating multiple sclerosis [134,135]. It downregulates the expression of the S1P1R receptor to inhibit the NF-kB pathway, which can alleviate [136,137]. Treatment with bortezomib alters S1PRI receptor activity in astrocytes leading to diminishing neuropathic pain [133]. Intrathecal administration of S1PR1 agonist leads to mechanical allodynia in wild-type mice and knocking out S1PR1 receptors in astrocytes does [138]. However, the effects of S1P1R inhibition on preventing and treating CIPN may not work in females [134]. More studies are required to further confirm the therapeutic effect of S1P1R antagonists.

### 5.2. Cannabinoids

Several animal studies have shown the correlation between endocannabinoid systems and peripheral neuropathy induced by several chemotherapy reagents. Both cannabinoid agonists and cannabinoid enzyme degradation inhibitors (fatty acid amide hydrolase and monoacylglycerol lipase) were efficacious in controlling CIPN. Treatment with cannabigerol, a bioactive compound from Cannabis, diminished the mechanical hypersensitivity by activating α2-adrenergic, CB1, and CB2 receptors in mice receiving cisplatin [139,140]. Administration of cannabidiol and anandamide prevents the development of neurotoxicity induced by paclitaxel via activating the serotonin 1A receptor and reducing the expression of toll-like receptor (TLR)-4 and Iba1 [141,142,143]. Some synthetic cannabinoids, GAT229, WIN55212, and PrNMI, can activate the CB1 receptors to allodynia induced by cisplatin [144,145,146]. Seminally, treatment with synthetic CB2 agonists, such as LY2828360, AM1710, JWH133, and MDA7, attenuates paclitaxel-induced mechanical and cold hypersensitivity by reducing the expression of TNF-α, brain-derived neurotrophic factor (BDNF), and MCP-1 in the lumbar spinal cord [146,147,148,149,150,151,152]. 

The transporters and enzymes, such as fatty acid amide hydrolase (FAAH), which are involved in cannabinoid receptor function, have been targets for discovering drugs for preventing and treating CIPN [97,98]. A few FAAH inhibitors, including ST4070, URB597, URB937, JZL184, and MJN110, have been tested in animal models of CIPN. Administration of these inhibitors reduces the mechanical hypersensitivity and spontaneous pain behavior evoked by cisplatin [153,154,155,156,157,158,159].

The analgesic effect of crude extract of Cannabis and some minor cannabinoids produced by Cannabis has also been studied in CIPN [160,161,162]. The supercritical fluid carbon dioxide extract of *Cannabis sativa* L. (Hemp) reduced the mechanical and thermal hypersensitivity by altering the neuroactive ligand–receptor interaction pathway, PPAR signaling pathway, and cAMP signaling pathway [162]. However, some minor cannabinoids, such as cannabinol (CBN), cannabidivarin (CBDV), cannabigerol (CBG), Δ8-tetrahydrocannabinol (Δ8-THC), and Δ9-tetrahydrocannabivarin (THCV), separated from Cannabis had a different analgesic effect. Only CBN was able to relieve the CIPN symptoms in a particular study [161]. 

Cannabinoids may enhance the analgesic effect of drugs for relieving the CIPN symptoms. Haddad et al. (2023) found that co-treatment with WIN55212 exhibited a great inhibitory effect on capsaicin-induced calcium responses which contributed to the development of nociception [163]. A combination of HU-210 (a CB1R agonist) and SNC80 (DOR agonist) reduced the allodynia in mice treated with paclitaxel [164]. CB2 agonist LY2828360 not only delays the development of neuropathy symptoms but also inhibits morphine tolerance in the CIPN model [152,165]. The combination of cannabidiol and mitragynine significantly reduces thermal hypersensitivity in mice treated with paclitaxel [166]. Some other compounds, including mitragynine, ART26.12, and hyperbaric oxygen, also prevent the CIPN symptoms by activating the cannabinoid receptors [167,168,169].

### 5.3. Histone Deacetylase 6 (HDAC6) Inhibition

Histone deacetylase 6 (HDAC6) is an enzyme that regulates multiple intracellular functions via deacetylate non-histone proteins [170]. Studies have found that HDAC6 inhibitors, such as ACY-1215, ACY-1083, and ACY738, can prevent and reverse the development of CIPN [40,171,172,173,174]. Treatment with ACY-1215 or ACY-1083 blocks the cisplatin-induced mechanical allodynia by preventing the loss of intraepidermal nerve fibers and mitochondrial damage in peripheral nerves and dorsal root ganglia neurons [40,171]. ACY-1215 and ACY-1083 can also increase the population of M2-macrophages to promote the production of IL-10 leading to a neuroprotective effect in the DRG neurons [172]. In addition, ACY-1083 enhances the tonic enkephalin-DOR (delta opioid receptor) signaling sensory neurons to prevent CIPN [173]. Those findings indicate that HDAC6 inhibitors could be promising drugs to prevent and treat CIPN.

### 5.4. Interleukin (IL)-10

Interleukin 10 is a classic anti-inflammatory cytokine produced by different types of immune cells [175]. It is a negative feedback regulator that regulates the inflammatory responses to different physiological and pathological conditions [176]. In the last few years, increased IL-10 has been found in different approaches to prevent and treat CIPN. Chemotherapy drugs, such as cisplatin and paclitaxel, can activate CD8+ T cells and CD4+ T cells or trigger receptors expressed on the myeloid cells 2 (TREM2)/DNAX-activating protein of 12 kDa (DAP12), signaling to increase production of IL-10 and leading to the resolution of CIPN [177,178,179,180,181]. Treatment with trimetazidine, inducible co-stimulatory molecule (ICOS) agonist antibody, or adenosine receptor (A3AR) agonist increases the production of IL-10 in the DRG to reduce the mechanical hypersensitivity induced by paclitaxel [44,182,183]. Interestingly, nasal administration of mesenchymal stem cells increases the production of IL-10 to reverse paclitaxel-induced neuropathy [184].

### 5.5. Transient Receptor Potential Vanilloid 1 and Ankyrin 1 (TRPV1 and TRPA1)

TRPV1 and TRPA1 are non-selective cation channels and are well-known nociceptive receptors for sensing thermal stimulation [185]. The GG-genotype of TRPV1 is associated with severe CIPN in patients with non-small cell lung cancer [186]. Administration of chemotherapy drugs can activate the TRPV1 and TRPA1 channels to induce neuropathy [187,188,189,190]. Studies have found that some potential treatments can reduce the CIPN by inhibiting the activities of TRPV1 and TRPA1. Antioxidants, such as henyl N-tert-butylnitrone and gergenin, reduce the activation of TRP channels to relieve the mechanical hypersensitivity in the animals receiving paclitaxel [187,191]. Intrathecal administration of D-series resolvin 5 (RvD5) reduces the mechanical allodynia induced by paclitaxel only in male mice, but not female ones [192]. Treatment with LPP1 and pregabalin attenuates thermal hypersensitivity, which is partially contributed by the inhibition of TRPV1 and TRPA1 [193]. Apolipoprotein A-I binding protein (AIBP) attenuates the toll-like receptor 4 (TLR4)- and TRPV1- induced pathways to prevent the development of CIPN [194]. 

### 5.6. Sterile Alpha and TIR Motif Containing 1 (SARM1) Inhibition

The sterile alpha and TIR motif containing 1 (SARM1) is an enzyme that degenerates axons by increasing intra-axonal calcium flux [195,196]. Pharmacological inhibition or genetic knockout SARM1 has shown protective effects on axons, blocking the development of CIPN. Several studies reported that irreversible SARM1 inhibitors or knockout SARM1 prevented chemotherapy drug (i.e., paclitaxel, vincristine, and bortezomib)-induced intraepidermal nerve fiber loss to maintain axonal function [197,198,199,200]. Knockout SARM1 can also reduce the changes in gene expression in the DRG to block the mechanical and cold hypersensitivity induced by oxaliplatin [201].

### 5.7. Herbal Medicines

Herbal medicines provide great resources for drug discovery [202]. Numerous herbs have been tested in preventing and treating CIPN. The decoction of Divya-Peedantak-Kwath (DPK) reduced inflammation and oxidative stress to prevent the allodynia and hyperalgesia induced by paclitaxel [203]. Goshajinkigan (GJG), a Japanese herbal medicine, prevented paclitaxel-induced neuropathy by inhibiting the activation of astrocytes in the primary sensory cortex [204,205,206]. Oral administration of ginger water extract increased the expression of 5-HT1A receptors, blocking oxaliplatin-induced cold and mechanical allodynia [207]. Danshen and its bioactive compounds, tanshinone IIA and cryptotanshinone, dose-dependently diminished oxaliplatin-induced CIPN [208]. Commiphora myrrha (CM) resin extract upregulates TRPV1 expression in the spinal cord to prevent the pathogenesis of paclitaxel-induced thermal hyperalgesia and mechanical allodynia [209]. Herbal prescriptions normally include several types of herbs. Some studies reveal that herbal prescriptions, such as SH003 and Siwei Jianbu, show neuroprotective effects, preventing CIPN symptoms [210,211,212].

### 5.8. Others

Stroke Homing peptide (SHp)-guided deoxyribonuclease 1 (DNase1) and inhibition of myeloperoxidase (MPO) or peptidyl arginine deiminase-4 (PAD4) can reduce chemotherapy-induced mechanical hyperalgesia and prevent the development of CIPN in mice model [213]. Inhibiting the CXCR1/2 signaling pathway using reparixin or ruxolitinib attenuated the development of allodynia induced by oxaliplatin, but not vincristine [214]. Nicotinamide Riboside (NR) is a vitamin B3 precursor of NAD+ which shows efficacy in suppressing tactile and cold hypersensitivity induced by paclitaxel in rats [215]. The NR blunts the loss of the intraepidermal nerve fiber. A high NAD+ level also helps with the deacetylation of alpha-tubulin in DRG via normalizing sirtuin Z [216]. Intraperitoneal injection with magnolin can reduce cold allodynia by inhibiting the activation of extracellular signal-regulated kinase (ERK) in mice with CIPN [217]. Oral treatment with antioxidants, such as N-acetylcysteine, α-lipoic-acid, Vitamin C, and Vitamin E, can dramatically inhibit the neuropathy and neuroinflammation induced by increased production of reactive oxygen species associated with oxaliplatin [218,219]. 

Numerous other potential targets have been discovered for treating CIPN. Peroxisome proliferator-activated receptor gamma (PPARγ) coactivator 1α (PGC1α) is important for maintaining the function of mitochondria and can reduce oxidative stress, leading to the prevention of CIPN [220]. Treatment with PPARγ activators, such as TZD-A1 and Ursolic acid, decreases paclitaxel-induced mechanical and thermal hypersensitivity [221,222]. Activation of adenosine monophosphate (AMP)-activated protein kinase (AMPK) can promote the expression of macrophage scavenger receptor A1 (SR-A1) or suppressors of cytokine signaling (SOCS) 3, which reduces CIPN in the mice model [223,224]. Vargas-Aliaga A. and colleagues have found that the development of CIPN could be contributed by the genetic polymorphisms of ATP binding cassette subfamily B member 1 (ABCB1, C1236T, and C3435T), but not Glutathione-S Transferase (GST) genes, in patients who received chemotherapy drugs [225,226]. Some other targets, such as the spinal neuronal microRNA-124 (miR-124), miR-3184-5p, Kinin B1 and B2 Receptors, and major histocompatibility complex II (MHCII), have shown activities that regulate the development of CIPN [227,228,229,230,231]. 

**Table 2:** Medications in preclinical evaluations to treat CIPN.

Intervention	Animal Model	Chemotherapy	Results	Reference
Amifostine	Mouse	oxaliplatin	Amifostine prevents mechanical hyperplasia and thermal allodynia development as well as protecting from neural hyperplasia and damage.	[232]
Anandamide	Rat	cisplatin	Anandamide can prevent mechanical allodynia induced by cisplatin	[143]
αO-Conotoxin GeXIVA[1,2]	Mouse	oxaliplatin	GeXIVA[1,2] can attenuate the development of oxaliplatin-induced CIPN	[233]
C781	Mouse	paclitaxel	Reverse the mechanical allodynia	[234]
Calmangafodibir	Mouse	oxaliplatin	Calmangafidipir can protect against mechanical allodynia and thermal hyperplasia induced by oxaliplatin	[79]
Cannabinoid	Rat	cisplatin	Significant reduction of mechanical allodynia threshold in rats treated with cannabinoids administered intraperitoneally or locally	[146]
Cannabigerol (CBG) and Cannabidiol (CBD)	Mouse	oxaliplatin	CBG, CBD, or a combination of both can reverse the mechanical hypersensitivity	[235]
CB2 agonist	Mouse	paclitaxel	Suppress allodynia induced by paclitaxel	[147]
Cannabigerol (CBG)	Mouse	cisplatin	Attenuates mechanical hypersensitivity	[139]
Cannabinoid enzyme degradation inhibitor	Mouse	paclitaxel	Suppressed mechanical and cold allodynia	[156]
	Rat	vincristine	Suppressed mechanical allodynia	[159]
	Mouse	cisplatin	Suppress mechanical allodynia, prevent mechanical hyperplasia	[158]
CGRP monoclonal antibody (ZR8 mAb)	Mouse & Rat	cisplatin	ZR8 mAb reduces mechanical hypersensitivity and thermal nociceptive sensitization induced by cisplatin	[236]
Glutathione	Mouse	oxaliplatin	GSH relieves neuropathic pain by chelating aluminum	[237]
Glycyrrhizic Acid	Mouse	paclitaxel	GA exhibits neuroprotective activity and attenuates the development of CIPN	[238]
Minocycline	Mouse	vincristine	Mechanical hypersensitivity reduction with no change in thermal threshold	[239]
Minocycline	Mouse	paclitaxel	Minocycline can prevent hypoesthesia and hyperesthesia induced by paclitaxel	[77]
Naringenin	Mouse	paclitaxel	Attenuate the neuropathic pain induced by paclitaxel	[240]
Nicotinamide riboside	Rat	paclitaxel	NR can suppress tactile and cold hypersensitivity without altering tumor growth	[215]
*Phlomidis radix* (*P. radix*) ethanol extract	Mouse	paclitaxel	*P. radix* and its bioactive compound, sesamoside, diminish the cold and mechanical pain	[241]
Phosphosulindac	Mouse	paclitaxel, vincristine, or oxaliplatin	PS dose-dependently relieves the allodynia in mice with CIPN	[242]
Polyvalent immunoglobulins	Mouse	vincristine or oxaliplatin	Attenuate tactile/cold hypersensitivity and nerve injuries	[243]
Prazosin + duloxetine	Rat	oxaliplatin	Combination of those drugs reduces paw withdrawal, but no improvement in allodynia or hyperalgesia	[244]
PNA6	Mouse	oxaliplatin	PNA6 relieves mechanical hypersensitivity	[245]
SS-20	Mouse	paclitaxel	Inhibit the development of CIPN	[246]
Synthetic peripherally restricted cannabinoids	Rat	cisplatin	Dose-dependently suppress mechanical and cold allodynia in local and systemic administration	[144]
Trimethoxyflavanone (Y3)	Mouse	paclitaxel	Y3 reduces the activities of DRG neurons to reduce the CIPN.	[247]

## 6. Discussion

In this paper, we have summarized the current information related to the pathophysiology and treatment of CIPN. We also discuss the clinical and preclinical trials that test potential approaches for CIPN management. 

The neuropathic pain induced by chemotherapy drugs has attracted a lot of interest in drug discovery. Cancer patients are treated with at least one type of chemotherapy reagent after surgically removing the tumor [248]. Thus, CIPN is predictable in cancer survivors and should be managed with standardized guidelines. Developing an individualized protocol should be considered to prevent and treat CIPN based on the chemotherapy agent(s) to be used. 

Several drugs, including Duloxetine, TCA medications, and ion channel modifiers, are used for CIPN treatment, even without FDA approval. Ion channel modifiers have also been shown to be successful in reducing CIPN. Even drugs exhibiting promising therapeutic effects still lack sufficient data to support their use for CIPN treatment. Several potential approaches have been tested in clinical trials. Cannabinoids have risen as a medication to treat emesis, nausea, and vomiting in cancer patients [249]. According to the findings from several clinical studies, the National Academies of Sciences, Engineering, and Medicine have confirmed the promising results of cannabinoids and cannabis in treating CIPN [42]. Non-opioid analgesics, metformin, minocycline, and Calmangafodipir appear to be effective in delaying the onset and reducing the intensity of CIPN symptoms in the acute and chronic phases of neuropathy. However, more clinical trials are required to confirm their therapeutic effects. The potential adverse effects of those reagents are also unclear. 

As discussed above, discovering drugs for preventing and treating CIPN has been a hot topic. A few directions could be considered. First, prevention and prophylaxis should be the focus and should be considered in order to reduce the burden on cancer patients who are already suffering. CIPN is an irreversible side effect [2]; thus, approaches for preventing the development of CIPN should be prioritized. Appropriate experimental design and large sample size are required to determine novel approaches for the prevention of CIPN [9]. Second, developing safer chemotherapy drugs with less risk of nerve injury should be focused on. Third, calcium and magnesium channels could be targets for discovering approaches to preventing CIPN [80]. Finally, the combination of pharmacological and nonpharmacological approaches should be applied to patients who have suffered CIPN to increase the efficacy of treatment and improve the quality of life. 

In conclusion, the information summarized in this paper provides a better understanding of the approaches and potential medications for treating CIPN. However, more studies are required to develop an efficient plan for the management of CIPN. Further, discovering approaches to preventing the pathogenesis of CIPN should be considered.

## Data Availability

No new data were created in this paper.
